# Target Therapy in Malignant Pleural Mesothelioma: Hope or Mirage?

**DOI:** 10.3390/ijms24119165

**Published:** 2023-05-23

**Authors:** Federica Borea, Marika A. Franczak, Maria Garcia, Matteo Perrino, Nadia Cordua, Ryszard T. Smolenski, Godefridus J. Peters, Rafal Dziadziuszko, Armando Santoro, Paolo A. Zucali, Elisa Giovannetti

**Affiliations:** 1 Department of Biomedical Sciences, Humanitas University, Via Rita Levi Montalcini 4, Pieve Emanuele, 20090 Milan, Italy; 2 Department of Medical Oncology, Amsterdam University Medical Centers, Location VUmc, Cancer Center Amsterdam, 1081 HV Amsterdam, The Netherlands; 3 Department of Biochemistry, Medical University of Gdansk, 80-210 Gdańsk, Poland; 4 Faculty of Experimental Science, Universidad Francisco de Vitoria, 28223 Madrid, Spain; 5 IRCCS Humanitas Research Hospital, Humanitas Cancer Center, Via Manzoni 56, Rozzano, 20089 Milan, Italy; 6 Department of Oncology and Radiotherapy and Early Phase Clinical Trials Centre, Medical University of Gdansk, 80-210 Gdańsk, Poland; 7 Fondazione Pisana per la Scienza, 56017 Pisa, Italy

**Keywords:** malignant pleural mesothelioma, target therapy, anti-angiogenic drugs, growth factors, mTOR, CDKN2A, NF2, BAP1, mesothelin

## Abstract

Malignant Pleural Mesothelioma (MPM) is a rare neoplasm that is typically diagnosed in a locally advanced stage, making it not eligible for radical surgery and requiring systemic treatment. Chemotherapy with platinum compounds and pemetrexed has been the only approved standard of care for approximately 20 years, without any relevant therapeutic advance until the introduction of immune checkpoint inhibitors. Nevertheless, the prognosis remains poor, with an average survival of only 18 months. Thanks to a better understanding of the molecular mechanisms underlying tumor biology, targeted therapy has become an essential therapeutic option in several solid malignancies. Unfortunately, most of the clinical trials evaluating potentially targeted drugs for MPM have failed. This review aims to present the main findings of the most promising targeted therapies in MPM, and to explore possible reasons leading to treatments failures. The ultimate goal is to determine whether there is still a place for continued preclinical/clinical research in this area.

## 1. Introduction

Malignant Pleural Mesothelioma (MPM) is a rare neoplasm, mainly related to environmental or occupational exposure to asbestos, with a long latency time (20 to 50 years) [1]. Typically, it is diagnosed in a locally advanced stage and therefore it is not eligible for radical surgery, but rather for systemic treatment. According to the WHO classification, there are three primary histological subtypes of this condition: epithelioid (accounting for 50–60% of cases), which usually has the most favorable prognosis; sarcomatoid (10% of cases), which is generally more aggressive and resistant to chemotherapy; and biphasic (30–40% of cases), which exhibits intermediate behavior [1]. The histological characteristics of MPM are closely associated with the process of epithelial-mesenchymal transition (EMT), in which epithelial cells acquire a mesenchymal phenotype, thereby increasing their potential for invasion and migration [2].

Chemotherapy with cis- or carboplatin and pemetrexed has been the only approved standard of care as first-line treatment for approximately 20 years, although it is associated with modest response outcomes and with a median overall survival slightly higher than 12 months [3,4]. 

No significant therapeutic advance has occurred until the introduction of immunotherapy and in particular of immune checkpoint inhibitors (ICIs), which act by interrupting the immune-suppressive interaction between checkpoint molecules expressed on tumor cells and their receptors on immune cells [1,5]. 

The recent phase III trial Checkmate-743 revealed that the combination of two ICIs, namely ipilimumab and nivolumab (which inhibit CTLA-4 and PD-1, respectively), had a statistically significant improvement on overall survival compared with pemetrexed and platinum chemotherapy (median overall survival (OS) of 18.1 versus 14.1 months, *p* = 0.002), especially for non-epithelioid subtypes [6]. Thus, this combination is now the recommended first-line treatment for MPM. 

However, MPM prognosis remains generally poor, usually not exceeding 18 months, and therapeutic options, particularly in second and subsequent lines of treatment, are still limited and have low effectiveness. Additionally, a relevant proportion of patients are primary refractory to immunotherapy, which further adds to the challenge of treating this condition [7]. 

Therefore, new pharmacological strategies are needed. In recent years, there has been a relevant improvement in our understanding of the molecular mechanisms underlying tumor biology, leading to the development of targeted agents that specifically target key pathways involved in several solid malignancies [8]. In the case of MPM, several pathways have been identified, including those related to growth factors, angiogenesis, cell cycle regulation, epigenetic modifications, DNA damage repair, and apoptosis [9]. However, despite promising preclinical results, most clinical trials evaluating potential targeted drugs have yielded unsatisfactory results.

The aim of the present review is to highlight the main molecular targets that have been evaluated in the context of MPM, along with the most promising targeted therapies and their relative findings. This review also aims to assess potential explanations for treatment failures and to consider whether further efforts in this area are warranted.

## 2. Main Molecular Targets

### 2.1. Growth Factor Pathways

**Figure 1:**
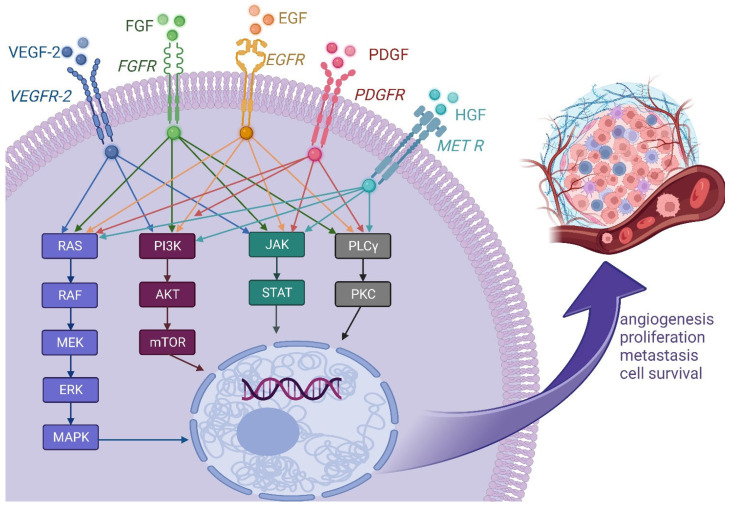
Upregulation of growth factors that activate the tyrosinase kinase receptors in MPM cells leads to the initiation of several pathways, which in turn modify gene transcription in the nucleus and provide cancer cells with many features increasing their aggressiveness. VEGF(R): vascular endothelial growth factor (receptor); PDGF(R): platelet-derived growth factor (receptor); EGF(R): epidermal growth factor (receptor); FGF(R): fibroblast growth factor (receptor); HGF hepatocyte growth factor; MET R: hepatocyte growth factor receptor; RAF: Rapidly Accelerated Fibrosarcoma; MEK: mitogen-activated kinase/ERK kinase; ERK: extracellular regulated kinase; MAPK: mitogen-activated protein kinase; PI3K: type I phosphoinositide 3-kinase; AKT: Protein kinase B; mTOR: mammalian target of rapamycin; JAK: Janus kinase; STAT: signal transducer and activator of transcription; PLCγ: phospholipase Cγ; PKC: protein kinase C.

As for many other malignancies, the importance of growth factor-related pathways in MPM development and progression is well-established, and their upregulation—predominantly mediated by tyrosine kinases (TKs)—has been identified and targeted [10,11]. To date, the vascular endothelial growth factor (VEGF) pathway has received the most attention, although the potential of targeting other pathways that contribute to angiogenesis, cell survival, growth, and proliferation—including those that depend on platelet-derived growth factor (PDGF), epidermal growth factor receptor (EGFR), fibroblast growth factor (FGF), and the MET receptor—have also been explored due to extensive crosstalk among various growth factor signaling pathways (Figure 1; Table 1), as is common with many other malignancies.

#### 2.1.1. Vascular Endothelial Growth Factor 

Preclinical and clinical evidence has largely demonstrated that angiogenesis represents a crucial step in MPM development, as it is necessary to supply oxygen and nutrients to the cancer cells. Since the early 2000s, it has been recognized that MPM cells are capable of producing growth factors that promote angiogenesis, particularly the VEGF, and of expressing high levels of the corresponding receptors, forming autocrine loops [12,13]. Physiologically, VEGF signaling is involved in the development and maintenance of vascular structures from embryogenesis to adult life [14]. Different isoforms of VEGF and two main tyrosine kinase receptors (VEGFR-1 and -2) are known, of which VEGRF-2 is a major mediator of biological effects. This receptor activates several mechanisms in endothelial cells promoting differentiation, survival, proliferation, migration, and angiogenesis. Two main pathways involved in promoting cellular growth and survival are Raf/MAPK and PI-3 kinase-Akt [15]. Moreover, by interacting with VEGFR2, VEGF activates the extracellular-signal-regulated protein kinases (ERKs) 1 and 2, through the activation of the Raf-1/MEK/ERK cascade and this is another crucial pathway for promoting angiogenesis [16]. Despite the formation of new blood vessels, these vessels in tumor tissue are typically abnormal in their structure, making the tumor tissue hypoxic and hard to access for cancer drugs [17]. Patients with MPM have higher levels of serum VEGF than individuals with other solid tumors or asbestos-related benign pleural diseases. Additionally, high levels of VEGF are associated with a worse prognosis [11,18]. As a result, targeting the VEGF pathway is a logical approach, and multiple inhibitors have been investigated.

One of the most studied anti-VEGF molecules is bevacizumab, a recombinant antibody able to neutralize all the isoforms of human VEGF. It was the first anti-angiogenetic to be FDA-approved for use along with chemotherapy [19]. Studies conducted on immunodeficient mice, orthotopically xenografted with MPM cells, have demonstrated a synergistic effect of bevacizumab and pemetrexed in inhibiting cell growth, suppressing pleural effusion, and prolonging survival [20]. Different clinical trials have subsequently evaluated the effectiveness of adding bevacizumab to chemotherapy in MPM patients. In a double-blind, placebo-controlled, randomized phase II trial, bevacizumab was combined with gemcitabine and cisplatin in unresectable chemo-naïve patients. The primary endpoint was progression-free survival (PFS), which did not differ significantly between the two groups of patients treated with bevacizumab or placebo (6.9 months vs. 6.0 months, respectively, *p* = 0.88). Additionally, no significant benefit was observed with bevacizumab in terms of improving OS [21]. Another single-arm phase II study that investigated the combination of bevacizumab with carboplatin and pemetrexed in a similar patient population also yielded negative results. The study aimed to achieve a 50% improvement in PFS compared to standard chemotherapy alone based on data from the literature (from 6 to 9 months), but the median PFS observed was 6.9 months. However, the combination therapy showed a median OS of 15.3 months [22]. In contrast, the Mesothelioma Avastin Cisplatin Pemetrexed Study (MAPS) yielded positive results. This large, open-label, randomized phase III trial enrolled 448 chemo-naïve patients, with 223 receiving pemetrexed and cisplatin plus bevacizumab (PCB) and 225 receiving chemotherapy alone (PC). The primary endpoint was the OS, which was improved in the PCB group compared with the PC one (median OS of 18.8 months versus 16.1 months, respectively; hazard ratio (HR) 0.77, *p* = 0.0167). Similarly, the median PFS was longer in patients receiving PCB than in those receiving PC (9.2 months vs. 7.3 months; adjusted HR 0.61, *p* < 0.0001). However, treatment with bevacizumab was associated with more severe toxicities (71% of G ≥ 3 adverse events (AEs) in the PCB group versus 62% in the PC group) and, as a result, a higher number of treatment interruptions (24.3% vs. 3%). Despite the exclusion of patients with significant cardiovascular comorbidities from the trial, a greater number of cardiovascular events, severe hypertension, thromboembolic, or hemorrhagic events (including a grade five brain hemorrhage) were reported in the PCB group [23]. Therefore, the MAPS trial is the first study demonstrating a survival benefit of an antiangiogenic drug combined with standard chemotherapy in well-selected patients with unresectable MPM eligible for bevacizumab, suggesting a new standard of care validated by the National Comprehensive Cancer Network and by French guidelines. However, because the trial did not include a comparison of bevacizumab with a placebo, and was not specifically designed as a registration trial, as well as due to the observed toxicity profile, health authorities worldwide have not approved this strategy.

Ramucirumab, a humanized monoclonal antibody binding VEGFR-2, was also tested in MPM. This treatment specifically targets the expression of VEGFR-2 on both MPM cells and macrophages, leading to the direct inhibition of tumor cell proliferation, as well as to reduced macrophage activity. This results in decreased tumor immune infiltration, and subsequently lowers cytokine and chemokine release, ultimately leading to reduced tumor growth and proliferation [24]. Studies conducted in animal models have demonstrated that combining chemotherapy with anti-VEGFR2 antibodies can improve the effectiveness of chemotherapy [25]. In line with these results, the RAMES study, a double-blind, randomized, placebo-controlled phase II trial, investigated the safety and efficacy of ramucirumab in combination with gemcitabine as a second-line treatment for 165 MPM patients. The primary endpoint of the study was OS, which was significantly longer in the experimental group compared to the control group, with a median of 13.8 months versus 7.5 months, respectively (HR 0.71, *p* = 0.028). The toxicity profile was overall manageable, despite a greater incidence of G3-4 AEs in patients receiving ramucirumab (44% vs. 30%), with neutropenia, hypertension, and fatigue as the most common events. In the experimental arm, thromboembolism was reported in 4% of patients versus 2% in the control arm, while no G3-4 hemorrhagic events or treatment-related deaths occurred in either treatment group [26].

#### 2.1.2. Platelet-Derived Growth Factor

PDGF is a glycoprotein with a strong mitogen effect on mesenchymal cells, physiologically involved in angiogenesis and tissue remodeling. Five isoforms of PDGF (PDGF-AA, -BB, -AB, -CC, and -DD), and two corresponding TK receptors (PDGFRα and PDGFRβ) are known [27]. Interestingly, PDGFs and VEGFs have been seen to have approximately 25% of their structure similar, with PDGF-C exhibiting an even greater similarity to VEGFs [28]. Binding of PDGF to its receptor triggers phosphorylation on specific tyrosine residues, resulting in the activation of multiple signaling pathways, particularly the PI3K/Akt and MAP kinase pathways, which stimulate cell growth, survival, and migration [28,29].

Compared to normal mesothelial cells, MPM cells express elevated levels of PDGFB chains and PDGFR β, and little or no expression of PDGFR alpha [30]. Autocrine and paracrine mechanisms, involving the interaction of PDGFB and PDGFR-β, have been proposed to be involved in the development and progression of MPM [31,32]. Additionally, Filiberti and collaborators observed a trend indicating that high serum PDGF levels might be associated with poorer overall survival in MPM patients, although this finding did not achieve statistical significance in a multivariate analysis [33].

Imatinib is a targeted drug that selectively inhibits PDGFR, c-kit, and bcr-abl. In vitro studies have demonstrated that it has a cytotoxic and pro-apoptotic effect on mesothelioma cells expressing PDGFR-β [34]. Additionally, PDGFR is involved in regulating interstitial fluid pressure, and imatinib can inhibit this signaling in the tumor microenvironment, leading to a reduction in interstitial fluid pressure and potentially enhancing drug delivery to the tumor tissues [35]. Nevertheless, when tested on pretreated or chemo-naïve mesothelioma patients in two phase II single-arm clinical trials, imatinib as single-agent showed disappointing results, with an overall response rate (ORR) of 0% in both studies [36,37]. Therefore, combination strategies were considered, given the observed synergistic effect in vitro between this drug and gemcitabine or pemetrexed [34]. Moreover, the combination of gemcitabine and imatinib was more effective than gemcitabine alone in a murine model. This combination was indeed shown to decrease tumor cell proliferation, increase apoptotic processes, inhibit tumor growth, and improve the survival of mice [38].

After conducting two phase I clinical studies to evaluate the safety and maximum tolerated dose of the combination in patients with different types of cancer [39,40], imatinib and gemcitabine were evaluated in MPM through a phase II single-arm trial on twenty-three pemetrexed-pretreated subjects. Although the trial attempted to select patients expressing PDFGRβ and/or cKIT by immunohistochemistry (IHC), it failed to meet its primary endpoint due to a lower-than-expected PFS rate at 3 months (39.1% compared to a pre-established target of ≥75%). The trial resulted in a median PFS of 2.8 months and a median OS of 5.7 months [41]. An additional phase I clinical trial was conducted on seventeen chemo-naïve MPM patients to assess the efficacy and safety of imatinib in combination with cisplatin and pemetrexed. However, out of the seventeen patients, only those six who had an optimal Eastern Cooperative Oncology Group (ECOG) PS and non-sarcomatoid histology, were able to complete all six planned treatment cycles. These patients reported a better PFS and OS, with 9.6 months and 22.4 months, respectively, compared to the entire study population, which had a PFS of 7.9 months and an OS of 8.8 months. However, the remaining patients had to discontinue treatment early, mostly due to disease progression, clinical deterioration, or poor tolerance [42].

#### 2.1.3. Epidermal Growth Factor Receptor

EGFR is a TK transmembrane protein, activated by a large family of closely related peptides, including epidermal growth factor (EGF) and transforming growth factor α (TGFα) [43]. Its activation promotes cell proliferation, motility, and invasion mainly via the RAS/RAF/MAPK pathway and the inhibition of apoptosis via the PI3K/AKT/mTOR pathway [44]. As observed for other growth factors, MPM cells seem to sustain an autocrine loop by the co-expression of both EGFR and TGFα [45]. IHC assays have shown that 44–97% of MPM patients exhibit an overexpression of EGFR, although its prognostic significance is still a matter of debate [46]. Targeting the EGFR pathway, which is routinely inhibited by monoclonal antibodies or TK inhibitors in other cancers such as colorectal and non-small cell lung cancer, has also been investigated in MPM [46]. Both gefitinib and PD153035, two anti-EGFR TKIs, demonstrated a promising cytostatic effect on four and six EGFR-expressing MPM cell lines, respectively [43,47]. However, in a single-arm phase II clinical trial on forty-three chemo-naïve mesothelioma patients (forty-two MPM and one peritoneal mesothelioma), monotherapy with gefitinib failed to meet the primary endpoint, because the percentage of patients alive and progression-free at 3 months was 40% and so lower than expected considering a historic control group from the Cancer and Leukemia Group B database [48]. A similar failure was observed in a subsequent single-arm phase II trial testing erlotinib, another EGFR TKI, in sixty-three chemo-naïve patients. In this case, the primary end point was 1-year survival, which was 40% with erlotinib versus 50% with standard cisplatin plus pemetrexed chemotherapy based on historical survival data [49]. Interestingly, both EGFR TKIs were ineffective despite high EGFR expression as assessed by IHC in most tumor samples [48,49]. Cetuximab, a chimeric mouse-human antibody directed against the extracellular domain of EGFR, was also studied in MPM. Of note, its mechanism of action differs from that of gefitinib and erlotinib, which act by competitively inhibiting the ATP-binding pocket of the receptor. Cetuximab impedes the binding of activating ligands, promotes internalization and degradation of EGFR, and as an IgG1 antibody, can trigger antibody-dependent cellular cytotoxicity (ADCC), a mechanism of cell-mediated immune defense [50,51]. Kurai et al. studied this drug in vitro and in vivo. On five MPM cell lines, no direct growth inhibitory effects were recorded, but a strong activation of ADCC was observed, and this was also seen in the case of weak EGFR expression (1+ by IHC). Moreover, this mechanism was enhanced when cetuximab was associated with IL-2, a lymphokine promoting the function of immune effector cells [51].

A follow-up study used an orthotopic mouse model, where the drug was directly administered into the thoracic cavity to evaluate its effectiveness. In this in vivo model, cetuximab was effective, resulting in a survival benefit for the treated mice. Interestingly, the co-administration of IL-2, although enhancing the inhibition of MPM tumor growth, did not increase the survival time compared to cetuximab alone [51]. Nevertheless, a phase II single-arm clinical trial evaluating cetuximab combined with standard platinum-based chemotherapy in a first line setting failed in reaching its primary end point, which was a PFS rate at 18 weeks. In particular, this trial was interrupted after a pre-specified interim analysis of the eighteen enrolled patients: thirteen of them (72%) were alive and progression-free at 18 weeks, falling short of the required fourteen or more patients. The trial showed an ORR of 44% (eight patients with partial response), a median PFS of 24 weeks, and a median OS of 49 weeks. The treatment was well-tolerated, with mild to moderate (G1-2) skin rash as the most common AE [52].

#### 2.1.4. Fibroblast Growth Factor

FGF is physiologically involved in several processes from embryonic development (i.e., organization of three germ cell layers, maturing of body axes, induction of organogenesis and morphogenesis) to adult life, when it contributes to tissue injury response, remodeling processes, and angiogenesis [53]. In humans, twenty-two FGF genes and four receptors (FGFR 1 to 4) have been identified [54]. Their interaction leads to the phosphorylation of specific tyrosine residues of the TK receptor as well as leading to the activation of various signaling pathways such as RAS-MAPK, PI3K-AKT, PLCγ, and STAT. These pathways, in turn, stimulate the transcription of different genes that regulate essential cellular processes such as proliferation, migration, and differentiation [54]. Aberrations in this pathway that lead to over-activation have been associated with drug resistance to several cytotoxic agents and TKIs [55]. High co-expression of multiple members of the FGF/FGFR family was observed in various MPM cell lines. However, no specific genetic aberrations were identified at high frequencies. Treatment with FGF2 induced EMT via MAP kinase signaling on these cell lines, which promoted proliferation, migration, scattering, and a spindle-shaped morphology [56,57]. On the other side, the inhibition of FGFRs or MAPK pathway seems to block signal transduction related to growth and survival and appears to induce a shift towards a more epithelioid gene expression and morphology in sarcomatoid cells [56,57]. In a non-randomized, open-label, phase Ib trial GSK3052230, a soluble fusion-protein with the capacity to bind FGFs and to inhibit FGF/FGFR signaling was administered in thirty-six patients affected by MPM as a first-line treatment in combination with cisplatin and pemetrexed [58]. Three dose levels of GSK3052230 (10 mg/kg, 15 mg/kg, and 20 mg/kg) were administered and, in thirty-one patients evaluable for disease response, an overall response rate of 39% and a disease control rate (DCR) of 86% were reported, with four patients maintaining disease control for over a year. Nine patients experienced serious AE (SAEs) and in six of them (mostly in the highest dose-level group) such SAEs were considered to be related to the study treatment, including a grade five toxicity (intestinal ischemia/perforation). It is interesting to note that GSK3052230, due to its structural features and inability to strongly bind specific FGFs, did not cause the typical AE observed with pan-FGFR kinase inhibitors, such as hyperphosphatemia, and tissue calcifications leading to retinal, nail, and skin toxicities [58]. However, to the best of our knowledge, there are no ongoing clinical trials investigating GSK3052230 despite its favorable clinical efficacy and acceptable toxicity profile at a dosage of 15 mg/kg [59].

#### 2.1.5. Hepatocyte Growth Factor HGF/MET Axis

The role of HGF and its TK receptor MET in tumor growth, metastasis development, and therapeutic resistance is well-established. In particular, HGF was initially characterized as a strong mitogen for hepatocytes, but was later recognized as a growth factor for several cell types and tissues. MET activation induces multiple signaling cascades, involving, among others, the RAS-MAPK and PI3K-AKT axis, and it is, therefore, able to promote cell proliferation, survival, and migration [60]. High levels of MET expression have been detected in MPM cell lines and tissues, where an autocrine loop seems to contribute to tumor angiogenesis [61,62].

Tivantinib is a MET inhibitor which is also capable of affecting microtubule polymerization [63]. The deregulation of the microtubule network identified in MPM makes this feature particularly intriguing [64]. In vitro, it has been demonstrated that the compound effectively suppresses cell growth in four MPM cell lines and exhibits a synergistic effect when used in combination with pemetrexed, enhancing its antiproliferative and pro-apoptotic actions [65]. In a phase I dose-escalation trial, tivantinib was tested in combination with carboplatin and pemetrexed as first-line treatment in 12 patients affected by MPM (*n* = 6) and advanced non-squamous non-small cell lung cancer (NSCLC) (*n* = 6), showing a 100% of disease control rate (DCR) [66]. Another different single-arm phase II trial investigated tivantinib monotherapy on 18 previously treated patients with pleural (*n* = 11) or peritoneal mesothelioma (*n* = 7), but it failed to achieve the primary ORR (0%). However, the peritoneal mesothelioma subgroup showed a DCR of 71% and a median OS of 22.2 months. No correlation was found between MET expression detected by IHC or mutation and disease control [67].

#### 2.1.6. Multi-Kinase Inhibitors

As previously mentioned, growth factors exert their effects by converging on common pathways that promote cell survival, growth, and proliferation. This observation may explain why some selective VEGF inhibitors have only shown partial success, while other selective TKIs have failed. It also supports the use of multi-kinase inhibitors. Several multi-kinase inhibitors, including valatanib [68], dasatinib [69], sunitinib [70,71], and sorafenib [72], which target VEGFR2 and PDGFRβ among other receptors, have been tested in small phase II single-arm clinical trials over the years. However, these compounds were not further evaluated due to their negative or not particularly promising results.

After showing a modest single-agent activity in pre-treated MPM [73], cediranib an oral VEGFR, c-kit, and PDGFR-β inhibitor, was evaluated in a double-blind, placebo-controlled phase II clinical trial in combination with platinum and pemetrexed chemotherapy as a first line treatment. The trial showed that cediranib met its primary endpoint, by improving PFS compared to placebo (7.2 months vs. 5.6 months, HR 0.71, *p* = 0.062), and it had a higher ORR in the experimental arm (50% vs. 20%, *p* = 0.006). However, no significant difference in OS was observed (10 months vs. 8.5 months in the placebo arm, HR 0.88, *p* = 0.28). Additionally, the toxicity profile was difficult to manage, with a higher incidence of grade 3–4 AEs in the cediranib arm (69% vs. 57%); especially diarrhea, hypertension, anorexia, dehydration, and weight loss. Due to the unfavorable safety profile and limited clinical benefit, no further studies with this drug have been conducted in MPM [74].

Nintedanib is a triple angio-kinase inhibitor targeting VEGFR, PDGFR, and FGFR. It showed promising results in vitro and in vivo, with additive effects on cell viability when added to cisplatin [75]. LUME-Meso is a randomized, double-blind phase II/III study to evaluate the efficacy of nintedanib plus first line chemotherapy with cisplatin and pemetrexed in unresectable MPM. In the phase II part, the primary end point was PFS, while OS and objective response were secondary endpoints. The addition of nintedanib to chemotherapy improved the median PFS by 3.7 months for the overall population (HR 0.54, *p* = 0.010), with a greater benefit in patients with epithelioid histology. Moreover, a positive trend in improving OS was observed in the experimental group, although the result was not statistically significant (median OS 18.3 months vs. 14.2 months in the control arm, HR 0.77, *p* = 0.319). Of note, stratifying by histology, the epithelioid subtype receiving nintedanib had a median OS of 20.6 months vs. 15.2 months in the placebo group [76]. After the positive results from the phase II portion of the trial, an amendment was made to exclude non-epithelioid histology for the confirmatory phase III part. A total of 458 patients were randomly assigned in a 1:1 ratio to either the nintedanib or placebo group, with PFS remaining as the primary endpoint and OS as a key secondary endpoint. Contrary to expectations, neither PFS nor OS showed any improvement in the experimental arm, with both groups having a median PFS of 6.8 months by independent central review (HR 0.99, *p* = 0.963), as well as a median OS of 14.4 months in the nintedanib group and 16.1 months in the placebo group (HR 1.12, *p* = 0.538) [77].

**Table 1:** Main clinical trials targeting molecular pathways in MPM.

Molecular Target	Drug	Mechanism of Action	Trial	Combination	Refs.
**VEGF**	Bevacizumab	binds to and neutralizes all human VEGF-A isoforms	double-blind, randomized phase II control: placebo	Gemcitabine Cisplatin	[21]
			single-arm phase II control: chemotherapy alone	Carboplatin Pemetrexed	[22]
			MAPS study open-label, randomized phase III control: chemotherapy alone	Cisplatin Pemetrexed	[23]
	Ramucirumab	selective against the extracellular domain of VEGFR-2	RAMES study randomized, double-blind phase II control: placebo	Gemcitabine	[26]
**PDGF**	Imatinib	selectively inhibits PDGFR	single-arm phase II pemetrexed-pretreated patients control: gemcitabine alone	Gemcitabine	[41]
			phase I	Cisplatin Pemetrexed	[42]
**EGFR**	Gefitinib	competitively inhibits the ATP-binding pocket of EGFR	single arm phase II control: Cancer and Leukemia Group B database	-	[48]
	Erlotinib		single arm phase II control: standard cisplatin plus pemetrexed chemotherapy database		[49]
	Cetuximab	blocks the binding of activating ligands of EGFR	single-arm phase II	Cisplatin/ Carboplatin Pemetrexed	[52]
**FGF**	GSK3052230	sequesters FGFs and blocks their ability to activate FGFR	non-randomized, open-label phase Ib	Cisplatin Pemetrexed	[58]
**HGF**	Tivantinib	MET inhibitor	phase I dose-escalation trial	Carboplatin Pemetrexed	[66]
			single-arm phase II trial	-	[67]
**Multi-target**	Cediranib	Inhibits VEGFR and PDGFR	double-blind phase II control: placebo with platinum-pemetrexed	Platinum Pemetrexed	[74]
	Nintedanib	Inhibits VEGFR, PDGFR and FGFR	LUME-Meso randomized, double-blind phase II/III control: placebo	Cisplatin Pemetrexed	[76,77]

Abbreviations. MAPS: Mesothelioma Avastin Cisplatin Pemetrexed Study; RAMES: RAmucirumab MESothelioma treatment; VEGF(R): vascular endothelial growth factor (receptor); PDGF(R): platelet-derived growth factor (receptor); EGF(R): epidermal growth factor (receptor); FGF(R): fibroblast growth factor (receptor); HGF hepatocyte growth factor.

### 2.2. Phosphatidylinositol 3-Kinase/Mammalian Target of Rapamycin/AKT

The activation of the PI3K/mTOR/AKT pathway promotes cell proliferation, growth, and survival, thereby playing a pro-oncogenic role in various types of tumors [78]. Hyperactivation of this pathway can be caused by various factors, including gain-of-function mutations in genes encoding its essential components, loss-of-function mutations in phosphatase and tensin homolog (PTEN, which typically functions as a suppressor of PI3K/AKT signaling), or constant activation of upstream growth factor receptor pathways [78].

Although everolimus had positive results in vitro in MPM cells [79], a single-arm phase II trial showed disappointing results. The trial involved 59 pretreated MPM patients, and a PFS rate of 29% was reported at 4 months (considering a prespecified primary endpoint of 50% or more), with an ORR of 2% and a median OS of 6.3 months [80].

On different MPM cell lines, drugs inhibiting both PI3K and mTOR markedly increased the inhibition of cell proliferation compared to selective inhibitors of PI3K or mTOR [81]. Samotolisib (LY3023414), a molecule targeting PI3K and mTOR, has been evaluated in a phase I cohort expansion study in 42 patients with pleural or peritoneal mesothelioma, refractory or ineligible for standard therapy. An ORR of 2.4% and a DCR of 43% were reported, with fatigue as the most common AE of every grade (G ≥ 3 in 10% of cases). Genetic data on tumor samples were available for 19 out of 42 patients, but no genetic alteration was found to be associated with antitumor activity [82].

### 2.3. Genomic Alterations

**Figure 2:**
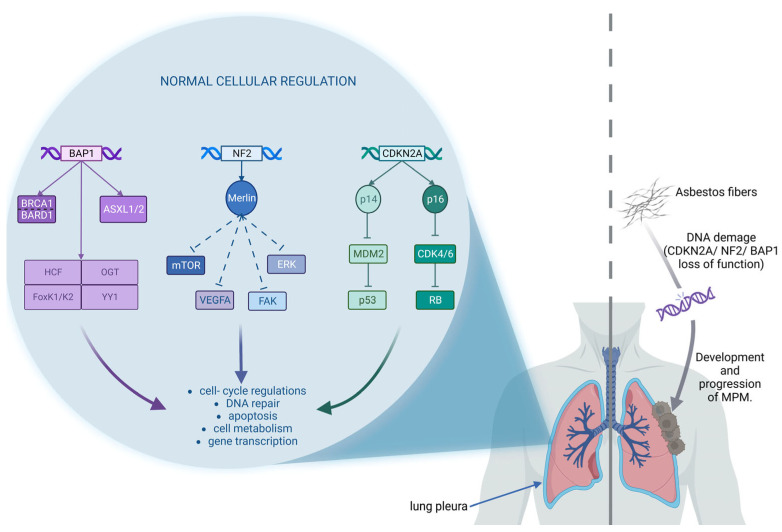
Three major genomic regulations in cells that are mostly suppressed in malignant pleural mesothelioma (MPM) induced by asbestos exposure. CDKN2A: Cyclin-Dependent Kinase Inhibitor 2; CDK4/6: cyclin-dependent kinases 4/6; RB: retinoblastoma protein; MDM2: Mouse double minute 2 homolog; NF2: neurofibromatosis 2; Merlin: Moesin ezrin radixin-like protein; ERK: extracellular signal-regulated kinase; FAK: Focal adhesion kinase; VEGFA: Vascular Endothelial Growth Factor A; mTOR: Mammalian target of rapamycin; BAP1: BRCA1—Associated protein1; ASXL1/2: Additional Sex Combs-Like 1/2; BRCA1: Breast cancer type 1 susceptibility protein; BARD1: BRCA1-associated RING domain protein 1; OGT: Protein O-GlcNAc transferase; HCF: Host cell factor; YY1: Yin Yang 1; FoxK1/K2: forkhead transcription factors.

Asbestos exposure can induce structural genomic rearrangements that often affect specific chromosome types and regions [83]. In particular, three tumor suppressor genes are frequently altered in MPM, thus losing their functions: Cyclin-Dependent Kinase Inhibitor 2/Alternative reading frame (CDKN2A/ARF) in about 80% of cases, neurofibromatosis 2 (NF2) in about 40% of cases, and BRCA1—Associated protein1 (BAP1) in around 30–60% of cases [84] (Figure 2). Gene therapy using viral or non-viral vectors to restore the functions of these genes could be a promising strategy, but limited efficacy has been observed in the few clinical trials conducted for MPM [85]. Another possible approach, studied more in recent years, aims to target the consequences of the above-mentioned genetic alterations (Table 2).

#### 2.3.1. CDKN2A/ARF and CDK4/6-Cyclin D1 System

The CDKN2A gene encodes for two proteins with a key role in cell-cycle regulations, p16ink4A and p14ARF. The first one inhibits the cyclin-dependent kinases (CDK) 4 and 6, preventing the phosphorylation and activation of the pro-oncogenic retinoblastoma protein (RB), while p14 inhibits mouse double minute 2 homolog (MDM2), preventing the ubiquitination and degradation of the onco-suppressor p53 [86]. Given the frequent occurrence of CDKN2A deletions in MPM, targeting CDK4/6 inhibitors could be a rational approach to achieve success in treating this type of cancer.

The Mesothelioma Stratified Therapy (MiST) trial is a phase II multi-arm trial that aims to enroll patients with relapsed MPM and assign them a specific study treatment based on the molecular profile of their tumor identified during a pre-screening phase. The trial consists of several arms (MiST 1 to 5), each designed as a single-arm, open-label trial (NCT03654833). Among these, MiST 2 evaluated the efficacy of the CDK4/6 inhibitor abemaciclib in second and subsequent lines on MPM patients negative for p16ink4A by IHC assay. The trial successfully met its primary endpoint of a 12-week DCR in at least eleven patients, with a DCR of 54% (fourteen patients, three with partial response and eleven with stable disease) among the twenty-six treated patients. Furthermore, a post-hoc exploratory analysis showed a median PFS of 128 days and a median OS of 217 days. Serious AEs occurred in 23% of patients, with one treatment discontinuation due to a non-neutropenic sepsis, and one fatal neutropenic sepsis was recorded [87].

#### 2.3.2. NF2 and Focal Adhesion Kinase

NF2 gene encodes for the Moesin ezrin radixin-like protein (Merlin), which acts as a negative regulator of several pathways, including mTOR and FAK ones [88]. FAK is a non-receptor cytoplasmic TK involved in cell proliferation, survival, invasiveness, and migration. FAK integrates signals from extracellular matrix proteins and cell membrane glycoproteins known as integrins. Due to its function and elevated expression in MPM, FAK was suggested as a potential therapeutic target, and initial in vitro findings about its inhibition were encouraging [89]. In two phase I studies enrolling pretreated patients with different solid tumors including MPM, the FAK inhibitor GSK2256098 alone [90] or in combination with the MEK inhibitor trametinib [91], showed a better efficacy and a longer PFS in patients with low expression of Merlin. However, the COMMAND (Control of Mesothelioma with MAiNtenance Defactinib) trial, a double-blind randomized phase II study evaluating the FAK inhibitor defactinib as maintenance after first-line chemotherapy, had negative results. A total of 344 patients affected by MPM, stratified for Merlin expression, were enrolled and randomized 1:1 to receive defactinib or placebo after at least four cycles of first-line chemotherapy. PFS and OS were co-primary endpoints, but neither was greater in the experimental arm than in the placebo arm, and both were without statistically significant differences according to high or low Merlin expression [92]. The investigators of the trial suggest that the failure of defactinib could be due to the modification of the immune microenvironment after chemotherapy, which may counteract the effects of the drug. FAK inhibitors are known to inhibit FAK-induced suppression of cytotoxic CD8+ T-cell activity, but after chemotherapy, an increase in programmed death ligand 1 (PD-L1) is induced, leading to immunosuppression [92]. Therefore, two clinical trials have been started to test defactinib in association with the PD-1 inhibitor pembrolizumab, but one was withdrawn for funding reasons (NCT04201145), while no news is available about the other (NCT02758587).

NF2 loss-of-function is also related to a deregulation of the Hippo pathway, involved in MPM cell proliferation and invasion in vitro [81]. Pevonedistat is a molecule inhibiting NEDD8 activating enzyme (NAE) and so attenuating the activation of yes-associated protein (YAP, a key component of the Hippo pathway) in NF2-mutant tumor cells. This drug is currently under evaluation for MPM in a two-cohort phase I/II trial, as monotherapy in NF2-mutant pretreated patients (cohort 1), or as combination therapy with cisplatin plus pemetrexed in first line treatment (cohort 2) (NCT03319537).

#### 2.3.3. BRCA1-Associated Protein 1 (BAP1) and DNA Damage System

The BAP1 gene encodes for a nuclear deubiquitinase, which is involved in numerous regulatory functions at the cellular level, influencing DNA damage response (DDR) and repair, gene transcription, cell-cycle control, cell metabolism, and apoptosis [93]. Germline or somatic BAP1 loss of functions is often involved in early development of MPM [93].

Due to the role of BAP1 in regulating BRCA1 expression (which is often low or absent in MPM patients) and its involvement in DNA double-strand break repair through homologous recombination, it is reasonable to explore the use of poly ADP-ribose polymerase (PARP) inhibitors in MPM [94], as is also suggested by in vitro studies [95].

MiST 1, an arm of the above-mentioned MiST trial, tested the PARP inhibitor rucaparib in second or subsequent lines in BAP1- or BRCA1-deficient mesotheliomas of any site. In twenty-six treated patients (twenty-three BAP1 deficient, thirteen BRCA1 deficient, ten both BAP1 and BRCA1 deficient), the administration of rucaparib was related to a 12-weeks-DCR of 58% (*n* = 15), meeting the primary endpoint of the trial. Six patients maintained DCR at 24 weeks. In addition, an ORR of 12% (*n* = 3, all partial response, PR) was observed, and post-hoc exploratory analyses showed a median PFS of 17.9 weeks and OS of 41.4 weeks. BAP1 and BRCA1 status by IHC did not appear to predict response to rucaparib. G3-4 AEs were recorded in nine out of twenty-six patients (35%), with upper respiratory tract infections and anemia being the most common. One patient discontinued treatment due to pericardial effusion, but no treatment-related deaths occurred [94].

A phase II trial investigated the efficacy of olaparib in previously treated mesothelioma patients (pleural or peritoneal) with a primary endpoint of ORR, based on the mutational status of DNA repair genes (germline or somatic). The trial enrolled a total of twenty-three patients (sixteen with pleural and seven with peritoneal mesothelioma), out of which eleven had a BAP1 mutation (eight somatic and four germline), and one had both somatic BAP1 and germline MRE11A mutations. The results showed only one patient with PR (4%), but with a disease control rate of 78% (eighteen out of twenty-three patients) for a duration of 6 weeks, and a median PFS and OS of 3.6 and 8.7 months, respectively. Germline BAP1 mutations were associated with lower PFS and OS compared to somatic ones (2.3 vs. 4.1 months and 4.6 vs. 9.6 months, respectively), but the trial results suggested that the effectiveness of olaparib was limited [96].

Similarly, another two-cohort phase II trial of niraparib in pretreated patients with solid tumors (including mesothelioma) and BAP-1 or other DDR genes mutations failed to meet the pre-specified efficacy threshold of ORR (NCT03207347). However, 78% of BAP1-mutant patients showed a clinical benefit [97].

UNITO-001-A is a another ongoing single-arm phase II study investigating the combination of niraparib and dostarlimab (an anti-PD1 monoclonal antibody) in homologous recombination deficient and PD-L1 positive MPM and NSCLC (NCT04940637). The same drug association is also being evaluated in the MIST 5 trial (NCT03654833). The rationale for this type of combination, especially in patients with DDR, is that PARP inhibitors may cause additional DNA damage, which has the potential to increase tumoral mutational burden by promoting neoantigen release and upregulating both interferons and PD-L1 expression, thus enhancing the activity of PD1/PD-L1 inhibitors [98].

### 2.4. Epigenetic Alterations

#### 2.4.1. Histone Deacetylase Inhibitors

Histone modifications due to alterations of the enzymes responsible for their deacetylation or acetylation can lead to gene silencing or inappropriate expression, respectively [99]. In vitro and in vivo preclinical data on histone deacetylase inhibitors in MPM supported their testing in clinical trials [100].

Vorinostat, a histone-deacetylase inhibitor acting by binding to the catalytic site of this enzyme, was tested in a double-blind phase III trial (VANTAGE-014) as second- or third-line treatment for MPM. A randomized trial enrolled a total of 661 patients, assigning them in a 1:1 ratio to receive either vorinostat or placebo, with the primary endpoints being OS, safety, and tolerability. The two groups showed a comparable tolerability profile, with similar rates of adverse events, except for a higher incidence of gastrointestinal AEs, anemia, and fatigue in the vorinostat group. Despite the three pre-planned interim analyses being positive, at the final data analysis, there was no statistically significant improvement in OS observed in the experimental arm compared to the placebo arm (30.7 weeks vs. 27.1 weeks, HR 0.98, *p* = 0.86) [101].

Similarly, the histone deacetylase inhibitor belinostat result was ineffective as monotherapy for pretreated MPM in a single-arm phase II trial. In this study, ORR was the primary endpoint, but it was not met in any of the thirteen enrolled patients in the pre-planned first-stage response analysis and, consequently, the accrual did not proceed to the next stage [102].

#### 2.4.2. Enhancer of Zeste Homolog 2 (EZH2)

(EZH2) is a regulator of gene expression. It participates in the histone methyltransferase polycomb repressive complex 2 (PRC2), which is involved in chromatin remodeling and maintenance of gene transcriptional repression by catalyzing the methylation of histone H3 on lysine 27 (H3K27me3) [103]. In MPM cell lines, BAP1 loss was observed to be related to increased EZH2 expression and enhanced repression of PRC2-regulated targets. In addition, BAP1-loss cells are more sensitive to EZH2 inhibitors [104]. Tazemetostat, which belongs to the same drug class, underwent testing in patients with relapsed or refractory MPM in a phase II single-arm trial consisting of two parts. The primary endpoint in part one was pharmacokinetic assessment, while in part two, it was 12-week DCR in BAP1 negative patients based on IHC, with a pre-set 35% threshold. A total of 74 patients were enrolled, with 13 in part one and 61 in part two, and 73 of them were BAP1 negative as determined by IHC. In part two, 54% of the 61 BAP1-loss patients met the primary endpoint with a 12-week DCR, while the ORR was 3%, with two PR observed at week 36 and week 42, respectively, and a median DOR of 30 weeks. The overall DCR for all 74 patients was 51%, with 35% having a reduction in tumor size, while the median PFS and OS were 18 weeks and 36 weeks, respectively [105]. In eight of the post-treatment cases, there was a significant reduction in H3K27me3 compared to baseline, while in the other two cases, there was an increase. It is interesting to note that all of the patients who had a reduction in H3K27me3 had stable disease at 12 and 24 weeks, while the two patients who experienced an increase had progressive disease at week 12. Additionally, there was a reduction in stromal and intratumoral B cells in nine and eight out of ten samples, respectively. Unfortunately, paired biopsies were not available for the two patients who achieved a PR [105]. 

**Table 2:** Main clinical trials targeting genetic and epigenetic targets in MPM.

Target	Drug	Trial	Refs.
**CDK4/6**	Abemaciclib	MiST2: single-arm, open-label, phase II	[87]
**FAK**	GSK2256098	phase I	[90]
		phase I, in combination with Trametinib (MEK inhibitor)	[91]
	Defactinib	COMMAND: double-blind, randomized, phase II	[92]
**PARP**	Rucaparib	MiST1: single-arm, phase IIa	[94]
	Olaparib	single arm, phase II	[96]
	Niraparib	two-cohort, phase II	[98]
**histone-** **deacetylase**	Vorinostat	VANTAGE-014: double blind, phase III	[101]
	Belinostat	single arm, phase II	[102]
**EZH2**	Tazemetostat	2-parts; single arm, phase II	[105]

*Abbreviations.* FAK: Focal adhesion kinase; COMMAND: Control of Mesothelioma with MAiNtenance Defactinib; PARP: poly ADP-ribose polymerase; EZH2: *enhancer* of zeste homolog 2; ADI-PEG20: Pegylated arginine deiminase; ADAM: Arginine Deaminase and Mesothelioma.

### 2.5. Mesothelin

Mesothelin is a membrane glycoprotein highly expressed in several malignancies, including ovarian cancer [106], pancreatic adenocarcinoma [107], and MPM, especially in epithelioid subtype [108]. Its physiological role is not completely understood, but in MPM it seems to promote tumor invasion and matrix metalloproteinase secretion [108].

Over the years, various immunotherapeutic approaches aimed at targeting mesothelin have been assessed. The initial one to undergo clinical trials was SS1P, an immunotoxin that comprised a murine anti-mesothelin antibody and a fragment of Pseudomonas exotoxin. Although it exhibited a favorable safety profile as a standalone therapy, its clinical efficacy was restricted mainly due to the emergence of neutralizing anti-drug antibodies (ADAs) [109,110]. Due to observed synergism between SS1P and chemotherapy in vivo, a phase I study was conducted to evaluate this strategy with pemetrexed and cisplatin as first-line treatment. Of the 20 MPM patients evaluated, a 60% ORR (12 patients, all PR, mostly at the MTD) and a 75% DCR (15 patients) were reported [111]. However, despite chemotherapy-induced myelosuppression, in 90% of patients ADAs were detected by cycle two, resulting in subtherapeutic SS1P concentrations [111]. Another trial evaluated the combination of SS1P with pentostatin and cyclophosphamide, two chemotherapeutic drugs that induced a selective lymphodepletion in vivo, and so prevent anti-immunotoxin antibody formation [112]. Indeed, the combination was found to effectively reduce and delay the development of ADAs when tested on ten patients with chemotherapy-refractory mesothelioma (eight pleural and two peritoneal). Among these patients, a durable PR (>14 months) was reported in three patients, and three others had SD [113].

LMB-100 is a more recent anti-mesothelin immunotoxin, with a less immunogenic structure compared to SS1P. In a phase I trial that involved dose-escalation and evaluated LMB-100 in twenty-five patients with solid tumors expressing mesothelin, there were no reported tumor responses. However, among the ten mesothelioma patients (three with pleural and seven with peritoneal mesothelioma), eight exhibited SD, while two patients with MPM had PD [114]. Despite expectations, all patients developed neutralizing antibodies to LMB-100 after repeated administrations, and its half-life was shorter compared to SS1P [114]. These findings, together with disappointing response rates, suggest a limited clinical efficacy of LMB-100 as a monotherapy. However, considering preclinical evidence of potential synergism with combination strategies [115], the trial was expanded to evaluate the maximum tolerated dose (MTD) of LMB-100 plus nab-paclitaxel in pretreated patients with pleural or peritoneal mesothelioma (NCT02798536). Additionally, following further preclinical data [116], another study evaluating intratumor LMB-100 injection plus ipilimumab (an anti-CTLA4 antibody) is currently ongoing (NCT04840615).

Amatuximab is a monoclonal antibody of the chimeric IgG1 kappa type that targets mesothelin and inhibits cell adhesion and stimulates cell lysis. A good safety profile of this drug as monotherapy was observed in a phase I trial on advanced mesothelin-expressing cancers, including MPM [117]. Consequently, amatuximab was tested in combination with cisplatin and pemetrexed in a single-arm phase II study as first line treatment on 89 patients affected by MPM. The study did not meet its primary endpoint, because a 6-months PFS lower than the pre-set target was reported (51.3% vs. 62%, respectively). Despite this, the combination showed an improvement in median overall survival compared to historical controls, with a DCR of 90% and an ORR of 39.8% (all partial responses), and it was generally well-tolerated [118]. A subsequent randomized phase II trial (ARTEMIS) evaluating cisplatin and pemetrexed with or without amatuximab in a placebo-controlled manner was terminated early due to a business decision (NCT02357147), and there are currently no ongoing clinical trials investigating this antibody [59].

Anetumab ravtansine (AR) is an anti-mesothelin antibody-drug conjugate, composed of a human antibody targeting mesothelin conjugated to the maytansinoid tubulin inhibitor DM4 [119]. In vitro it showed robust cytotoxicity on cells expressing mesothelin and in vivo it inhibited tumor growth, also exhibiting a bystander effect on near mesothelin-negative malignant cells [119]. In a phase I dose-escalation and dose-expansion study in pretreated patients with different malignancies, among mesothelioma patients a 31% ORR and a 75% DCR were reported at the maximum tolerated dose. Interestingly, higher mesothelin expression was found in those having a greater drug effect [120]. A phase Ib trial was conducted to evaluate the efficacy and safety of AR in combination with cisplatin and pemetrexed in pretreated patients with mesothelin-expressing NSCLC (one patient) and epithelial pleural or peritoneal mesothelioma (sixteen patients). Out of the sixteen evaluable patients, a 50% ORR (all PR) was observed, and the combination was found to have a manageable safety profile [121]. The results of a phase II trial (ARCS-M) evaluating AR as a second-line therapy for MPM have been recently published. The trial included 248 subjects with mesothelin-overexpressing tumors who were randomized in a 2:1 ratio to receive either AR or vinorelbine, with PFS by centralized review as the primary endpoint. Unfortunately, no statistically significant difference was observed between the experimental and standard treatment arms in terms of PFS or OS (median PFS of 4.3 months vs. 4.5 months, HR 1.22, *p* = 0.86; median OS of 9.5 months vs. 11.6 months, HR 1.07, *p* = 0.66). However, in a post hoc analysis, it was observed that patients in the AR group with mesothelin expression in the second or higher quartile had longer PFS and OS compared to those in the first quartile. This effect was not observed in the vinorelbine group [122]. Another phase I/II trial testing AR plus pembrolizumab is currently ongoing (NCT03126630).

Lastly, this review will not delve into cancer vaccines and Chimeric Antigen Receptor (CAR)—T cell therapy, which are more recent strategies aimed at inducing an immune response against mesothelin-expressing cells, as they are part of the field of immunotherapy [123].

### 2.6. Arginine

Arginine is an essential amino acid for cell survival and in mesothelioma, its metabolism is often disrupted due to the decreased or absent levels of arginine succinate synthetase 1 (ASS1), particularly in the sarcomatoid and biphasic subtypes. As a result, therapeutic strategies that induce arginine depletion can result in a synthetic lethality effect [124].

Pegylated arginine deiminase (ADI-PEG20), a cloned enzyme able to degrade this amino acid into citrulline and ammonia, was studied as a potential new treatment in different solid tumors, including MPM [125]. In the Arginine Deaminase and Mesothelioma (ADAM) phase II trial, sixty-eight ASS1-deficient (as determined by IHC) MPM patients were enrolled and randomized 2:1 to receive ADI-PEG20 or best supportive care (BSC) as first or second line treatment. The primary endpoint of this trial was PFS, which was found to be higher in the ADI-PEG20 treatment group compared to the control group (with a median of 3.2 vs. 2.0 months, respectively, HR 0.56, *p* = 0.03) [126]. Therefore, in a subsequent phase I dose-escalation trial, the combination of ADI-PEG20 with cisplatin and pemetrexed was tested on nine ASS1-deficient chemo-naïve patients (five MPM and four NSCLC). Among MPM patients, four PR and one SD were observed, without dose-limiting toxicities [127]. In a dose-expansion cohort, thirty-two chemo-naïve patients received the same drug combination, using ADI-PEG 20 at the recommended dose of 36 mg/m^2^. A good tolerability and a promising clinical activity were confirmed, with a DCR of 93.5% (twenty-nine out of thirty-one patients) and an ORR of 35.5% (eleven patients, all PR). A median PFS and OS of 5.6 and 10.1 months, respectively, were also reported [128]. In order to elucidate the mechanisms behind ADI-PEG 20 resistance, a subsequent biopsy was suggested at the time of disease progression, and six patients agreed to undergo the procedure. Compared to the baseline, the samples collected at disease progression revealed a marked infiltration of macrophages, which is consistent with a potential stromal-mediated pathway of resistance. Additionally, PD-L1 upregulation and the presence of CD3+ T lymphocyte clusters were observed in two cases, indicating a potential advantage of combining ADI-PEG 20 with immunotherapeutic agents [128]. Lastly, The ATOMIC Meso trial, a randomized double-blind phase II/III study evaluating the addition of ADI-PEG 20 to pemetrexed and cisplatin, has recently been completed, but the results have not yet been released (NCT02709512).

## 3. Potential New Targets

As our understanding of the molecular mechanisms behind MPM advances, potential new targets are emerging. In particular, AXL, lactate dehydrogenase (LDH), and glucose transporter 1 (GLUT-1) have shown promise in preclinical studies, but there is currently no clinical data available.

### 3.1. AXL

The TAM group is a small family of three tyrosine kinase receptors (TKRs), comprehending TYRO-3, AXL, and MER, which are physiologically involved in several processes including cellular proliferation, adhesion, migration, survival, and regulation of inflammatory cytokine release [129,130].

AXL and its major ligand, GAS6, are overexpressed and activated in many solid tumors, including lung cancer, pancreatic cancer, and MPM, correlating with poor prognosis and promoting invasiveness, angiogenesis, EMT phenotype, and drug resistance [131,132]. Song and collaborators found that the pro-oncogenic role of AXL is justified, at least in part, from the induction of a p53 loss-of-function through a negative regulation of this gene transcription. On the other hand, AXL inhibition by the small molecular drug R428 (bemcentinib) led to decreased viability, migration, and invasion of MPM cells [133]. Moreover, targeting AXL seems to promote chemotherapy sensitivity, as observed in vitro by Oien and collaborators. In particular, they found that cisplatin and pemetrexed treatment causes the formation of reactive oxygen species (ROS), inducing AXL phosphorylation and activation, and that AXL inhibition by bemcentinib, administered before or after chemotherapy, has a high synergistic effect [134]. Currently, the third arm of the MiST trial is testing bemcentinib in association to pembrolizumab in pretreated MPM patients (NCT03654833).

### 3.2. Lactate Dehydrogenase (LDH)

LDH is an enzyme that converts pyruvate to lactate, a process that is highly active in the Warburg effect, where cancer cells switch their energy production from oxidative phosphorylation to anaerobic glycolysis [135]. The Warburg effect provides cancer cells with many advantages, including resistance to treatment and the ability to metastasize [136]. In addition, the serum level of LDH is a well-known marker of cancer progression and lack of treatment efficacy and high levels are associated with poor overall survival [137,138]. LDH is particularly important in highly hypoxic tumors, which are characterized by low oxygen availability and other metabolic substrates [136]. As a result, LDH has become an interesting target in various malignancies, including mesothelioma. Several LDH inhibitors, such as galloflavin [139], GNE-140 [140], and oxamate [141] have shown promising preclinical results in different cell lines. However, only n-hydroxyindole (NHI) inhibitors of LDH have been tested in MPM, showing a disturbance in cellular metabolism, increased sensitivity to gemcitabine and pemetrexed, and therefore hold promise for future clinical trials [142,143,144].

### 3.3. Glucose Transporters (GLUT)

GLUT) are a large family of membrane carriers responsible for the internalization of glucose, a major substrate for energy production [145]. High expression of GLUT type 1 (GLUT-1) was observed in many cancers, including basal-like breast cancer, gastric cancer, and lung cancer [146,147,148], and was correlated with tumor aggressiveness and shorter patient survival [149]. A meta-analysis showed that GLUT-1 is also a good marker to differentiate MPM from reactive mesothelioma cells [150]. Nowadays, several GLUT inhibitors such as WZB117, BAY-876, and STF-31, have been preclinically tested, and the results have shown that GLUT inhibition sensitizes cells to anti-cancer treatment, blocks cell growth and glycolysis, and induces apoptosis [151,152,153]. On MPM cells, GLUT-1 inhibition by salicylketoxime derivatives had a cytotoxic effect and led to energy imbalance [144]. However, none of these inhibitors have been tested in clinical trials yet.

## 4. Discussion

The enthusiasm for molecularly targeted therapies has steadily increased in recent years. However, in mesothelioma, despite promising results from in vitro and in vivo models, clinical trials in this area have often been disappointing. To understand the issue, several critical points need to be highlighted.

Firstly, there seems to be a significant gap between preclinical models and human subjects in mesothelioma, which is not as apparent as in other solid tumors. Mesothelioma is known for its molecular and clinical heterogeneity, which means that the outcomes observed in the laboratory may not be consistent with real-life situations. Mice are the most common animal models used for experiments, but they do not always provide reliable results, due to both technical and biological factors. In mesothelioma, where the tumor typically spreads locally, intrapleural drug administration is preferred. However, the procedures for appropriate MPM xenotransplant and treatment administration are complex in mice. Moreover, the interplay between the tumor cells and the microenvironment cannot be overlooked, and despite high homology between the human and mouse genomes, the characteristics of the peritumoral stroma may not be entirely identical or reproducible [154].

It is also important to take into account the unique genetic and pathogenetic features of MPM. Asbestos, a carcinogen that does not cause mutations, has been linked to MPM, resulting in a lower tumor mutation burden in MPM compared to other solid tumors [155]. The genes most frequently involved are tumor suppressors, which can be inactivated by genomic aberrations or epigenetic mechanisms due to alterations in histone-regulating proteins. As a consequence, several intracellular pathways (such as PI3K/AKT/mTOR or Hippo) are deregulated, without structural alterations in the growth factor receptors normally activating them, although the presence of autocrine loops involving growth factors are also well known for MPM [84]. The extent of functional dysregulation of these pathways in MPM tumor biopsies is difficult to measure. The combination of these factors explains the lack of success of many TKIs tested in MPM, including EGFR inhibitors. In all clinical trials involving these drugs, the EGFR gene status, and potential alterations in its downstream effectors (such as KRAS, BRAF, and PI3KCA), were not evaluated [48,49,52]. Hence, it is possible that inappropriate patient selection took place, given that, although high levels of EGFR expression were observed in IHC assessments, activating mutations or amplifications in the EGFR gene, which are typically associated with the efficacy of anti-EGFR TKIs, are uncommon in MPM [156,157]. Similarly, the presence of activating mutations in the genes encoding for the downstream effectors of EGFR could explain an intrinsic resistance to cetuximab, as is already known in colorectal cancer [158]. An additional mechanism of resistance to TKIs is related to the frequent loss, in MPM, of the tumor suppressor gene PTEN. This results in a lack of regulation and continuous activation of the PI3K/AKT/mTOR pathway and justifies the ineffectiveness of TKIs, considering that they act upstream of it [159]. Nevertheless, the limited single-agent clinical efficacy of the mTOR inhibitor everolimus [83], as well as of the mTOR and PI3K inhibitor samotolisib [85], highlights once again the presence of an extensive crosstalk between the different signaling intracellular pathways promoting cancer cell growth and survival.

In conclusion, it may be necessary to reconsider the notion that targeting a single factor can effectively manage MPM, and instead focus on designing combination strategies that work synergistically at multiple levels. Recent research has emphasized the importance of the tumor microenvironment and the immune system in MPM pathogenesis and resistance to treatment, which presents a strong rationale for combining target therapy and immunotherapy. However, some patients may not respond to immunotherapy alone, making the identification of predictive biomarkers crucial for directing treatment decisions and maximizing efficacy while minimizing toxicities and costs. The completion of the MiST trial (NCT03654833), which proposes a molecular-based therapeutic approach, is highly anticipated, and the results of its arms testing pembrolizumab plus bemcentinib (MiST 3), atezolizumab plus bevacizumab (MiST 4), and dostarlimab plus niraparib (MiST 5) will provide valuable insights. Although validation in larger studies is necessary, positive results could establish the role of targeted therapy in well-selected patients and bring us closer to personalized medicine for MPM.

## Data Availability

Not applicable.

## Abbreviations

(S)AE: (serious) adverse event, ADA: anti-drug antibody, ADAM: Arginine Deaminase and Mesothelioma, ADCC: antibody-dependent cellular cytotoxicity, ADI-PEG20: pegylated arginine deiminase, AR: anetumab ravtansine, ASS1: arginin succinte synthetase 1, BAP1: BRCA1—Associated protein1, BSC: best supportive care, CAR: chimeric antigen receptor, CDK4/6: cyclin-dependent kinases 4/6, CDKN2A: Cyclin-Dependent Kinase Inhibitor 2, COMMAND: Control Of Mesothelioma with MAiNtenance Defactinib, DCR: disease control rate, DDR: DNA damage response, ECOG: eastern cooperative oncology group, EGF(R): epidermal growth factor (receptor), EMT: epithelial mesenchymal transition, ERK: extracellular regulated kinase, EZH2: enhancer of zeste homolog 2, FAK: Focal adhesion kinase, FGF(R): fibroblast growth factor (receptor), GLUT-1: glucose transporter type 1, HGF: hepatocyte growth factor; HR: Hazard ratio, ICI: immune checkpoint inhibitor, IHC: immunohistochemistry, JAK: Janus kinase, LDH: lactate dehydrogenase, MAPK: mitogen-activated protein kinase, MAPS: mesothelioma avastin cisplatin pemetrexed study, MDM2: Mouse double minute 2 homolog, MEK: mitogen-activated kinase/ERK kinase, Merlin: moesin ezrin radixin-like protein, MET R: hepatocyte growth factor receptor, MiST: mesothelioma stratified therapy, MPM: malignant pleural mesothelioma, MTD: maximum tolerated dose, mTOR: mammalian target of rapamycin, NAE: NEDD8 activating enzyme, NF2: neurofibromatosis 2, NSCLC: non-small cell lung cancer, ORR: overall response rate, OS: overall survival, PARP: poly ADP-ribose polymerase, PC: pemetrexed cisplatin, PCB: pemetrexed cisplatin bevacizumab, PDGF(R): platelet derived growth factor (receptor), PD-L1: programmed death-ligand 1, PFS: progression-free survival, PI3K: type I phosphoinositide 3-kinase, PKC: protein kinase C, PLCγ: phospholipase Cγ, PRC2: polycomb-repressive complex 2, PTEN: phosphatase and tensin homolog, Raf: rapidly accelerated fibrosarcoma, RAMES: RAmucirumab MESothelioma, RB: retinoblastoma protein, ROS: reactive oxygen species,.STAT: signal trans-ducer and activator of transcription, TGFα: transformig growth factor alfa, TK(R): tyrosine kinase (receptor), VEGF(R): vascular endothelial growth factor (receptor), YAP: yes-associated protein 1.
